# Whole-Embryo 3D Quantification Reveals Flexible Cellular Scaling and Conserved Tissue Architecture in *Xenopus* Species

**DOI:** 10.64898/2026.06.16.732511

**Published:** 2026-09-16

**Authors:** Hugo Mauricio, Mariia Diakova, Max Brambach, Cora Anderson, Kseniya Petrova, Cecilia A. De Araujo, Iva Simeonova, Geneviève Almouzni, Leonid Peshkin, Jose G. Abreu

## Abstract

How embryos with markedly different absolute sizes and cell numbers establish comparable tissue organization during development remains a fundamental question in developmental biology. To address this question, we compared two closely related *Xenopus* species that differ substantially in embryonic size, *Xenopus laevis* and *Xenopus tropicalis* . We generated a whole-embryo quantitative 3D atlas of cell allocation, spatial organization, and mitotic dynamics at key time points between gastrulation to tailbud stages. Using tissue clearing, and 3D imaging we tracked single-nucleus coordinates across developmental milestones to resolve how body plans adapt to organismal scale. We show that embryonic scaling is not achieved through simple proportional changes in cell number. Instead, the smaller *X. tropicalis* embryo is characterized by a distinct high-density tissue organization associated with a persistently higher mitotic index (∼1.4-fold higher than in *X. laevis* at both gastrula and tailbud stages). Across development, this is accompanied by a near-doubling of cell number in *X. tropicalis* without a proportional increase in embryo volume. We quantify tissue organization and find species-specific cellular architectures during gastrulation that largely converge by the tailbud stage. At this stage, homologous tissues display broadly similar structural profiles despite persistent differences in embryo size, cell number, and density. Together, our findings reveal that closely related vertebrate embryos can follow distinct cellular organization trajectories while converging toward comparable tissue architecture, providing a quantitative framework for understanding robust body plan formation across divergent physical scales.

**Key Findings:**

- *The number of cells in X. tropicalis increase* s by nearly double between the gastrula and tailbud stages, even though the volume of the embryo does not increase accordingly.
- *X. laevis has a mitotic index that is about 1.4 times lower than that of X. tropicalis* at both the gastrula and tailbud stages.
- Systematic differences in tissue density are observed among the germ layers within the embryo (consistently in both species), with the neuroectoderm being among the most densely packed and the endoderm among the least dense.
- Cell number, density, and structure vary substantially among germ layers during gastrulation; however, these differences between germ layers diminish as development progresses.
- In the tailbud stage the homologous tissues in *X. laevis* and *X. tropicalis* show generally similar structural arrangements even though there are continuous differences in embryo size and cell number.

## Full Text Availability

The license terms selected by the author(s) for this preprint version do not permit archiving in PMC. The full text is available from the preprint server.

